# Outcome in Women with Traumatic Brain Injury Admitted to a Level 1 Trauma Center

**DOI:** 10.1155/2014/263241

**Published:** 2014-08-03

**Authors:** Elaine de Guise, Joanne LeBlanc, Jehane Dagher, Simon Tinawi, Julie Lamoureux, Judith Marcoux, Mohammed Maleki, Mitra Feyz

**Affiliations:** ^1^Neurology and Neurosurgery Department, McGill University Health Centre, 1650 Cedar Avenue, Montreal, QC, Canada H3G 1A4; ^2^Psychology Department, University of Montreal, C.P. 6128 Succ. Centre-Ville, Montreal, QC, Canada H3C 3J7; ^3^Traumatic Brain Injury Program, McGill University Health Centre, 1650 Cedar Avenue, Montreal, QC, Canada H3G 1A4; ^4^Physical Medicine and Rehabilitation Service, McGill University Health Centre, 1650 Cedar Avenue, Montreal, QC, Canada H3G 1A4; ^5^Social and Preventive Medicine Department, University of Montreal, 7107 Avenue du Parc, Montreal, QC, Canada H3N 1X7

## Abstract

*Background*. The aim of this study was to compare acute outcome between men and women after sustaining a traumatic brain injury (TBI). *Methods*. A total of 5,642 patients admitted to the Traumatic Brain Injury Program of the McGill University Health Centre-Montreal General Hospital between 2000 and 2011 and diagnosed with a TBI were included in the study. The overall percentage of women with TBI was 30.6% (*n* = 1728). Outcome measures included the length of stay (LOS), the Extended Glasgow Outcome Scale (GOSE), the functional independence measure instrument (FIM), discharge destination, and mortality rate. *Results*. LOS, GOSE, the FIM ratings, and discharge destination did not show significant differences between genders once controlling for several confounding variables and running the appropriate diagnostic tests (*P* < 0.05). However, women had less chance of dying during their acute care hospitalization than men of the same age, with the same TBI severity and following the same mechanism of injury. Although gender was a statistically significant predictor, its contribution in explaining variation in mortality was small. *Conclusion*. More research is needed to better understand gender differences in mortality; as to date, the research findings remain inconclusive.

## 1. Introduction

Differences have been found in the brains of men and women with regard to weight, neuronal density, and brain structure. More specifically, some studies have shown a larger volume of the hypothalamus and overall white matter in men whereas women's brains have been found to have more cortical gray matter, greater volume in regions associated with language function, greater volume of the hippocampus, and greater volume of white matter associated with interhemispheric connectivity [1, 2]. Based on these findings, it is reasonable to hypothesize that men's and women's brains may react to and recover from a traumatic brain injury (TBI) differently. Thus, TBI studies should examine the genders separately.

Men are twice as likely to experience a TBI as women [3, 4]. Results found in the literature on brain injury are therefore more likely linked to men than women yet generalization to women has always been implicit. Some earlier studies however did show a difference in the mortality and morbidity rates between genders but others did not. Discrepancies are still present in the literature in regard to mortality and morbidity following TBI and results of studies differ depending on whether or not covariables, such as age, TBI severity, or presence of multiple trauma, have been controlled for [5, 6].

In a prospective cohort of patients with moderate and severe TBI followed for a period of 3.5 years, mortality was 1.28 times higher in females than males, with the greatest difference being in deaths after discharge which was 2.14 times higher. Controlling for age, admission Glasgow Coma Scale score, penetrating as compared to blunt injury, and presence of multiple trauma, females were 1.75 times more likely to die of their brain injury than males. Furthermore, females were 1.57 times more likely to experience poor outcomes than males [5]. Other studies have also reported that women were less likely to survive following their TBI [6].

In contrast, other investigations have reported that female gender is not an independent risk factor for in-hospital mortality after TBI [5, 7]. A study done by Leitgeb and colleagues did not show a significant difference among TBI groups. Hospital mortality was 39.6% for females and 32.5% for males and the rates of unfavorable outcome were 58.7% for females and 53.4% for males [8]. Similar tendencies have been reported in a recent study done by Gao and Jiang [9] in an acute care setting. In this research, mortality for male and female patients with TBI was 7.48% and 7.22%, respectively, with corresponding unfavorable outcome being 16.05% and 17.23%. However, controlling for variables seems to affect results of this type of analysis as shown in a study done by Ng and colleagues [10]. In their work, females had a significantly higher mortality and poorer outcome compared with males but this difference was no longer significant when covariables (presence of multiple injuries, postresuscitation pupil abnormalities, and Glasgow Coma Score) were controlled for.

The inconsistencies in the current literature on TBI gender differences lead to the need to pursue the effort to better understand the relationship between gender and TBI variables. Introducing different demographic and clinical variables such as age and TBI severity in the analysis and controlling for these variables may help obtain a better comprehensive picture. Thus, the goal of the current investigation was to compare demographic, clinical, and psychosocial characteristics between men and women who sustained a mild to severe TBI and to look at differences in short-term outcome assessed in a Level 1 Trauma Center. Different outcome measures were analyzed including length of stay (LOS), scores on the Extended Glasgow Outcome Scale (GOSE) and on the functional independence measure instrument (FIM), discharge destination, and mortality rate. Results of these analyses will help to better identify the specific needs of patients with TBI admitted to acute care with regard to gender and to better plan future services for this population.

## 2. Methods

### 2.1. Subjects

A total of 5,642 patients admitted to the Traumatic Brain Injury Program of the McGill University Health Centre-Montreal General Hospital between 2000 and 2011 and diagnosed with a TBI by a physician were included in the study. Approval for this retrospective study was granted by the research ethics board of the MUHC-MGH. The overall percentage of women with TBI was 30.6% (*n* = 1728).

### 2.2. Demographic, Clinical, and Preinjury Variables

All data were collected by an experienced clinical manager retrospectively from a registry, from administrative TBI files and from medical files completed by physicians and allied professionals. Demographic characteristics analyzed were* age* (ordinal variable) and* level of education* (elementary, high school, college, and university). Moreover, the* trauma etiologies* included the following categories: motor vehicle accident, work accident, assault, and falls.* Level of alcohol upon admission* was also collected. The* injury severity score (ISS)*, the* Glasgow Coma Scale (GCS) scores*, and* the presence of multiple trauma (polytrauma)* were noted. The GCS score upon admission to the emergency room was used to determine severity of the TBI. A GCS score of 13–15 represents a mild TBI and that of 9–12 represents a moderate TBI, while a score of 3–8 indicates a severe TBI. A* complexity score* was computed by adding the number of complicating factors (comorbidities). These included a positive brain CT scan, past medical history of substance abuse, prior neurological condition (epilepsy, previous stroke, and brain tumors), intellectual impairments, severe behavioral issues, psychiatric diagnosis, dementia, suicidal attempts, and homelessness. This data was gathered systematically for each patient by an experienced social worker involved in the TBI team who conducted semistructured interviews with the patient or relatives. More specifically, medical, cognitive, and psychological antecedents were investigated with the patient, the family, or the family physician if need be. Dichotomized analyses (presence or absence) were carried out on these variables. Each variable, when confirmed, received a score of one and each variable was rated equally. All CT scans of the head were viewed by a neurosurgeon. A score of one (presence) was attributed when a CT scan done in the first 48 hours was read as positive by a neurosurgeon. Data to determine preinjury alcohol use were based on the use of alcohol one month prior to the TBI. Patients included in the group of preinjury alcohol abuse had a heavy alcohol consumption (more than 14 drinks a week for men and more than 7 drinks a week for women) at least in the last month prior to the TBI [11]; this information was confirmed by the patient, family member or relative, and/or patient's family physician. Information concerning prior neurological condition, intellectual impairments, severe behavioral issues, psychiatric diagnosis dementia, suicidal attempts, and homelessness was ascertained by the social worker. The complexity score was a cumulative score related to the number of patient antecedents; the higher the score, the higher the number of premorbid antecedents which were interpreted as a more complex situation in regard to comorbidities potentially influencing recovery from the TBI. This* ad hoc* score is used only in-house and has not been validated previously.

### 2.3. Outcome Variables

*Length of stay (LOS)* corresponded to the number of days the patient remained hospitalized in the acute care setting from admission to discharge.* The Extended Glasgow Outcome Scale* (GOSE) [12] score was also collected from the TBI program registry. Scores were determined at discharge from the acute care setting by the interdisciplinary team involved in the patient's care. The GOSE assesses global outcome. On this scale, scores of 7 or 8 correspond to good recovery referring to normal participation in social, vocational, and physical life. Scores of 5 or 6 indicate moderate disability describing the patient who is independent but physically or cognitively disabled and requiring an altered physical, social, psychological, or vocational environment for participation. Patients with severe disabilities receive scores of 3 or 4 and are totally dependent in managing a normal or modified environment whereas a score of 2 corresponds to a vegetative state reflecting total dependency with no awareness of the environment. Patients who died received a score of 1.

The* functional independence measure* (FIM) instrument was used to measure global functional levels [13]. The instrument is an 18-item 7-point scale, with increasing values indicating greater level of independence. Three ratings were used in this study. The first was a global rating including the 18 items for a total ranging from a minimum of 18 to a maximum of 126. The second was a physical rating including those items related to activities of daily living, continence, and mobility with a range of 13 to 91. The last rating was the cognitive domain consisting of social interaction, problem solving, memory, expression, and comprehension and ranged from 5 to 35. The FIM rating was given by the interdisciplinary team prior to discharge within no specific time frame. After 2007, the FIM license was not renewed, and the FIM instrument was no longer used by the team.

*Discharge destinations* included home without rehabilitation services, home with outpatient rehabilitation, inpatient rehabilitation, long-term care placement, and death.

### 2.4. Statistical Analysis

Descriptive statistics are presented using means ± standard deviation, medians and ranges for numerical variables, and proportions for categorical variables. Bivariate associations with gender were examined using Student's *t*-test (with or without Satterthwaite correction for unequal variances) for numerical variables with symmetric distributions, Kruskal-Wallis tests for ordinal variables or numerical variables with severe asymmetry, and chi-square tests for nominal variables. Ordinal (for GOSE and FIM scores) and logistic (for discharge location) regressions were used for predictions while controlling for the following confounding variables: age, year of accident, GCS score, ISS score, primary language, leaving against medical advice, and mechanism of accident. Once controlling for all these confounding variables and running the appropriate diagnostic tests, gender was added to determine if its contribution was statistically significant. To determine the usefulness of the independent variables in the predictive model, the Lacy coefficient of determination [14] was used which, contrary to other pseudo-R2, can be interpreted as the percentage of variation in the dependent variable explained by the independent variables. All tests were done at the 0.05 level of significance using Stata 12.1 (StataCorp, Texas).

## 3. Results

### 3.1. Demographic, Clinical, and Preinjury Variables

Since this was a retrospective study, some variables had more missing values than others. Overall, most variables had less than 5% missing values and these were assumed to be missing at random. The percentage of missing values is given in Tables 1 and 2. Level of education had a large proportion of missing values (72.2%) and was therefore not included in the predictive analyses. The complexity score variable (computed by the count of comorbid conditions) was not collected before 2004. Between 2004 and 2011, 36.1% of the subjects had missing complexity data and this variable was not included in the regression analyses. FIM ratings were collected between 2000 and 2007. During these years, 22.5% of the FIM values were missing and were assumed to be missing at random. For the GOSE scores, 6.4% of values were missing and those were also assumed to be missing at random.

**Figure 1:**
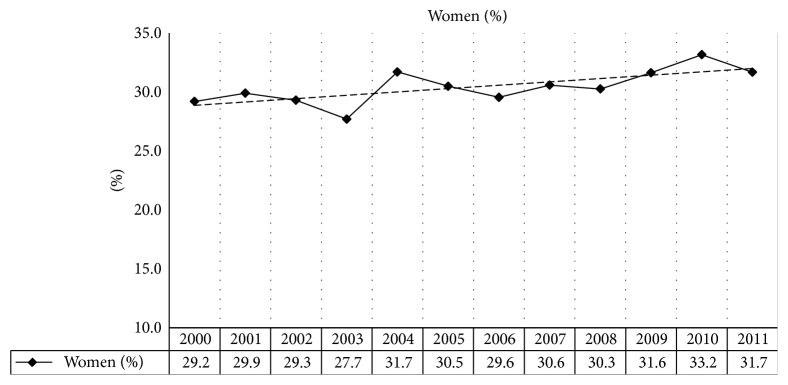
Trend in the proportion of women with TBI per year (*n* = 5642).

Figure 1 shows that the proportion of women with TBI was trending upwards over the years from 2000 to 2011. As shown in Table 1, women were significantly older than men by an average of 8 years and had a significantly higher education level (although one should be wary of this conclusion considering the high rate of missing values for education level). Women were less frequently involved in work accidents (0.9%) or in an assault (4.0%) compared to men (6.6% and 13.3%, resp.). The proportion of patients with mild TBI was significantly lower in the male (68.9%) group compared to the female group (73.9%). In addition, women had significantly lower ISS scores compared to men although the clinical significance of a 1-point average difference on the ISS scale is not clear. The complexity of the cases as measured by the number of comorbid conditions was not significantly different by gender.

### 3.2. Outcome Variables

#### 3.2.1. Length of Stay (LOS)

Bivariately, LOS (see Table 2) was not significantly different between genders. After controlling for confounding variables, the difference in LOS between genders was still not significant (*P* = 0.212).

#### 3.2.2. Extended Glasgow Outcome Scale (GOSE)

Table 2 shows the proportion of TBI cases per gender group in each of the GOSE categories at discharge. Bivariately, there was an association between gender and GOSE, with men having a slightly higher (better) score than women. However, after controlling for confounding variables in an ordinal regression, gender was no longer a significant predictor of GOSE outcome (*P* = 0.081).

#### 3.2.3. Functional Independence Measure (FIM)

Bivariately, (see Table 2) men had significantly higher scores (97.2 ± 30.1) compared to women (93.1 ± 30.5). Because of severe nonnormality, the FIM score was recoded into 7 equal categories of 20 units from 0 to 140 and an ordered logistic regression was done to predict the FIM score category controlling for confounding variables.  Table 3 gives the results of this ordinal regression. As shown, the odds of being in a higher FIM score category (better function) increased slightly from year to year. The odds of being in a higher FIM score category were lower for those having had a motor vehicle accident compared to those having suffered a fall. The odds of being in a higher FIM category decreased for those with a higher ISS score and increased for those with a higher GCS score. The odds of being in a higher FIM score category were lower as age increased and higher for those who left AMA. Controlling for all these factors, the odds of being in a higher FIM score category were lower for women; that is, a man would have a higher level of function at discharge from acute care compared to a woman of similar age and with a similar injury (severity and mechanism). Gender was not a statistically significant predictor of functional independence when using the Bonferroni correction. The model in Table 3 had a Lacy R2 of 23.2%, whereas a model without the gender variable had a Lacy R2 of 23.1%.

#### 3.2.4. Destination at Discharge

Bivariately (see Table 2), discharge destination was significantly different by gender. The various destinations were separated into indicators to determine how gender was associated with each one of them when controlling for confounding variables. In the cases of discharge home and discharge to outpatient rehabilitation, gender was not a significant predictor after controlling for confounding factors (*P* = 0.162 and *P* = 0.103, resp.).

A similar multiple logistic regression was performed to predict discharge to inpatient rehabilitation. The odds of being discharged to inpatient rehabilitation were higher for motor vehicle accident victims compared to fall victims and for those with higher ISS scores. The odds of going to inpatient rehabilitation increased with age and for those leaving AMA. After controlling for all these factors, the odds of going to inpatient rehabilitation were marginally higher for women compared to men of similar age and injury characteristics but the coefficient did not reach the level of significance corrected for multiplicity of tests. The regression model had a Lacy R2 of 8.0%, whereas a model without the gender variable had a Lacy R2 of 7.9%.

After controlling for confounding variables, there was no association between gender and being discharged to long-term care (*P* = 0.796).

#### 3.2.5. Mortality

A logistic regression was performed controlling for confounding factors. Leaving AMA was not included in this regression because none of those leaving AMA expired. The results, shown in Table 4, indicated that, for everything else being equal, the odds of dying decreased from year to year and increased with age. Having a higher ISS score increased the risk of mortality as did having a more severe TBI (a lower GCS score). The odds of dying were significantly lower for women compared to men of similar age, trauma etiology, and injury characteristics. Although gender was a statistically significant predictor, its contribution in explaining variation for mortality was small. The model in Table 4 had a Lacy R2 of 31.0%, whereas a model without the gender variable had a Lacy R2 of 30.7%.

## 4. Discussion

The aim of the present study was to compare acute outcome between men and women who sustained a TBI by controlling for different factors such as age and TBI severity.

Regarding the demographic and clinical variables of this cohort, 30.7% were females and the prevalence of work or assault related trauma was lower in this group which is in keeping with previous findings [3, 4]. Women tend to be less involved in work where there are greater risks of physical injury (such as in the construction industry) and are less frequently involved in physical altercations than men. Level of education was also higher for women (more years of schooling) but there were several missing data for this variable. Previous studies [15–17] demonstrated that educational level was related to functional outcome of patients with TBI. Unfortunately, because of the large number of missing values for level of education this variable could not be controlled in these predictive analyses. Age, however, was controlled as a covariable in the present study; the proportion of women in the older groups was higher than that in the younger group. Age is an important variable to consider in the analyses of differences in outcome between genders. In fact, it is well documented that age has been considered as a variable influencing the outcome of TBI. Many previous studies have reported that older patients have less favorable outcome than their younger counterparts [18–20] and older patients generally show a lower level of independence in daily activities after TBI [19–21]. Finally, the current study showed a difference in injury and TBI severity, with women showing lower ISS scores and higher GCS scores. Again, these factors were statistically controlled for in the analyses because of their impact on acute outcomes [17, 22].

With respect to the findings on acute outcome between the genders, there was a difference in level of independence (FIM rating) upon discharge from acute care despite the fact that there was no difference in LOS between men and women and no difference in global outcome (GOSE) after controlling for confounding variables. However, this difference did not reach the corrected level of significance after controlling for confounding variables such as age and TBI severity. Inconsistencies are also present in previous research that compared the relationship between gender and functional outcome. For example, a study by Doninger and colleagues [23] reported better community integration for women but other investigations have reported the opposite results with worse functional outcome for the group of women with TBI [7, 24] or no difference among groups [6, 25, 26]. A study done by Bazarian and colleagues revealed similar results with patients having suffered from mild TBI. They showed that males and females did not significantly differ with respect to the odds of poorer outcome as defined by the number of days to return to normal activities or the number of work days missed. However, there was a significant difference in postconcussive score and the results indicated significantly higher odds of poorer outcome in relation to this measure for females after controlling for appropriate confounders [27]. The results in the current study for functional outcome once again provide an unclear picture since the analyses did not show significant differences in functional outcomes between men and women when using a corrected level of significance for multiplicity of tests. However, discrepancies found in the literature on TBI as well as the discrepancy in outcome results in the study at hand may be related to the tools or instruments used to assess the outcome. No difference between men and women might be found when using a global measure of outcome (level of disability) but a tool measuring physical and cognitive functional outcome might better discriminate the two groups. In the context of an acute care setting, the FIM instrument has been demonstrated to be a useful, practical, and simple tool to assess initial disability and residual limitations [28, 29]. Regarding discharge destination as a tool to assess outcome, a previous study looking at discharge outcome showed that women fared less well after TBI and were significantly more likely than men to be sent to long-term care facilities rather than home settings, controlling for age, injury severity, mechanism of injury, and comorbidities [30]. In the present investigation, the difference in functional outcome was not reflected in discharge destinations. However, women showed marginally higher odds for being discharged to inpatient rehabilitation. The reasons why women were referred more frequently to inpatient rehabilitation or long-term care settings may be related to the fact that they presented with greater functional limitations following TBI, as shown with the lower scores upon discharge on the FIM instrument. A different hypothesis on availability of caregiver or other support could be entertained regarding this finding. It has been found that women who suffer from brain injury are less likely to have a spouse than men, leaving them with limitations but without home support [31]. Men, with functional limitations on the other hand, may be less likely to be referred to inpatient rehabilitation or to long-term care facilities because of the social or family support that they have available to them following their accident. It is probable that if they have a spouse, this person will act as a caregiver to provide the care and help they require so that a safe discharge home could be considered. In fact, in Canada, there are many more women who are devoted to a loved one by being a caregiver. A higher proportion of family/friend caregivers aged over 45 years are women (56.5%) compared to men (43.5%) [32] and caregivers are more often women compared to men even following a TBI [33]. In the present study no data were available in the registry regarding the presence of caregiver or other support.

The findings of this research paper suggest that women had less chance of dying than men of the same age and the same TBI severity with the same etiology of trauma in the same year of occurrence. Mortality differences have also been found in a study done with stroke patients, with women having a better 1-year survival rate after a stroke [34]. The hypothesis to explain this difference is related to progesterone's neuroprotective effect in TBI [35]. The animal and human brain injury literature suggests a possible female gender advantage in recovery from TBI based on the theory that the presence of progesterone has a neuroprotective effect following TBI [36, 37]. In these studies, female gender was independently associated with reduced mortality and less complications after TBI. The findings regarding gender differences in mortality after neurological insult need to be considered cautiously since there remains contradiction in the literature. For example, a recent study done by Lorenzano and colleagues (2013) with 45,079 patients treated with intravenous alteplase found no gender difference in the rate of symptomatic intracerebral hemorrhage but a significantly higher likelihood of functional independence at 3 months in men and a higher mortality in women when compared with men [38]. Moreover, results regarding one type of neurological condition cannot be generalized to another.

There were limitations to this study. This being a retrospective chart review, there is no control over the confounding variables except for statistical control. Moreover, this chart review included cases from only one hospital and thus does not reflect the situation in other centers.

In conclusion, several questions and contradictions remain regarding the reasons for the difference in mortality between men and women following a TBI. Could hormonal or functional or structural brain differences or even cerebral plasticity following TBI contribute to this disparity? Continued research on the role of gender in TBI outcome is necessary using appropriate statistical approaches, controlling for several confounding variables, and running the appropriate diagnostic tests.

## Figures and Tables

**Table 1:** Descriptive statistics for demographic and accident variables by gender.

Variables	Women	Men	Test (*P* value)
Age (missing: 0.1%)			
Mean (SD) Median (interquartile range)	57.2 (23.6) 59.5 (35.3–79.0)	49.2 (21.2) 47.9 (30.0–66.6)	*t*_3000df_ = 12.110 (*P* < 0.001)
Education (missing: 72.2%)			
Elementary	11.6%	12.1%	*χ* ^2^_3df_ = 13.623 (*P* = 0.003)
High school	41.7%	50.7%	
College	16.1%	13.7%	
University	30.6%	23.5%	
Etiology (missing: 0.0%)			
Fall	55.7%	49.8%	*χ* ^2^_3df_ = 216.797 (*P* < 0.001)
MVC	39.3%	30.3%	
Work accident	0.9%	6.6%	
Assault	4.0%	13.3%	
TBI severity (missing: 1.5%)			
Mild	73.9%	68.9%	*χ* ^2^_2df_ = 17.129 (*P* < 0.001)
Moderate	9.6%	10.1%	
Severe	16.5%	21.0%	
Injury severity scale (ISS) (missing: 5.6%)			
Mean (SD) Median (interquartile range)	22.9 (10.3) 24 (16–27)	23.9 (10.4) 25 (17–29)	*t*_5324df_ = 3.338 (*P* = 0.001)
Complexity (2004–2011) (missing: 36.1%)			
Mean (SD) Median (interquartile range)	1.45 ± 0.94 1 (1-2)	1.49 ± 1.00 1 (1-2)	*z* = 0.672 (*P* = 0.502)

**Table 2:** Distribution of outcomes by gender.

Variables	Women	Men	Test (*P* value)
Length of stay (missing: 0.3%)			
Mean (SD) Median (interquartile range)	15.00 (19.92) 9 (4–18)	14.84 (19.23) 8 (3–19)	*z* = 1.734 (*P* = 0.083)
GOSE (missing: 6.4%)			
Lower good recovery	65 (4.0%)	220 (6.0%)	*z* = 3.791 (*P* < 0.001)
Upper moderate disability	567 (35.2%)	1485 (40.4%)	
Lower moderate disability	541 (33.6%)	1040 (28.3%)	
Upper severe disability	208 (12.9%)	360 (9.8%)	
Lower severe disability	48 (3.0%)	91 (2.5%)	
Vegetative state	24 (1.5%)	52 (1.4%)	
Death	159 (9.9%)	423 (11.5%)	
Discharge destination (missing: 0.8%)			
Home	687 (40.1%)	1674 (43.1%)	*χ* ^2^_5df_ = 42.687 (*P* < 0.001)
Outpatient rehab	165 (9.6%)	507 (13.1%)	
Inpatient rehab	473 (27.6%)	920 (23.7%)	
Long-term care	128 (7.5%)	190 (4.9%)	
Transfer to acute	86 (5.0%)	144 (3.7%)	
Death	173 (10.1%)	449 (11.6%)	
FIM (2000–2007) (missing: 22.5%)			
Mean (SD) Median (interquartile range)	93.1 (30.5) 105 (76–116)	97.2 (30.1) 110 (84–119)	*z* = 3.931 (*P* < 0.001)

**Table 3:** Results of logistic regression predicting FIM score category (*n* = 4532).

	Odds ratio	Standard error	*z*	*P* > |*z*|	[95% conf. interval]
Year	1.047	0.015	3.21	0.001	1.018–1.076
Age	0.986	0.002	−5.83	<0.001	0.981–0.991
ISS	1.008	0.005	1.54	0.123	0.998–1.017
GCS	1.088	0.016	5.59	<0.001	1.056–1.121
DC AMA	0.272	0.140	−2.52	0.012	0.099–0.749
French language	1.299	0.128	2.63	0.009	1.069–1.578
Etiology (compared to falls)					
MVC	0.854	0.097	−1.39	0.164	0.684–1.066
Work related	1.262	0.244	1.20	0.229	0.864–1.843
Assault	1.113	0.173	0.69	0.492	0.821–1.508
Female	0.791	0.088	−2.10	0.036	0.636–0.984

**Table 4:** Results of logistic regression predicting mortality (*n* = 4532).

	Odds ratio	*P* > |*z*|	[95% conf. interval]
Year	0.919	<0.001	0.886–0.953
Age (square of age)	1.0004	<0.001	1.0004-1.0005
ISS (1/*√*ISS)	8.71*e* − 09	<0.001	1.54*e* − 10–4.94*e* − 07
GCS	0.748	<0.001	0.728–0.769
Etiology (compared to falls)			
MVC	0.774	0.068	0.588–1.019
Work related	1.159	0.630	0.635–2.116
Assault	1.027	0.913	0.639–1.651
French speaking	0.867	0.232	0.686–1.095
Female	0.662	0.002	0.510–0.860
